# Can Industrial Helmets Protect the Head in Simulated Falls and Trips?

**DOI:** 10.1007/s10439-026-04013-z

**Published:** 2026-02-13

**Authors:** Rachel Jia Ying Tan, Xiancheng Yu, Mazdak Ghajari

**Affiliations:** 1 HEAD Lab, Dyson School of Design Engineering, Imperial College London, London, SW7 2AZ UK; 2 School of Mechanical, Aerospace and Civil Engineering, Sheffield, S1 3JD UK

**Keywords:** Industrial helmet testing, Brain injury, Falls and trips, Safety helmet, Head impact condition, Work-related injury

## Abstract

**Purpose:**

Falls and trips are a leading cause of work-related traumatic brain injuries, yet the protective performance of industrial helmets in such scenarios remains poorly understood. This study assesses the effectiveness of different industrial helmet designs under impact conditions representative of falls and trips.

**Methods:**

Six industrial helmets with different designs were tested. Four were suspension-based models compliant with EN 397, including two versions of the same model, one with and one without the rotation reduction system, MIPS. Two additional helmets were foam-based, meeting both EN 397 and EN 12492 standards. Helmets were dropped onto angled anvils at different speeds and impact locations to simulate trips and falls. Tests were conducted on two surface types: P80 abrasive papers and roof shingles. The new EN 17950 headform was used.

**Results:**

Helmet performance varied by design and impact condition. Foam-based helmets offered better protection against impacts than suspension-based helmets, which showed greater sensitivity to impact location. Front impacts near the rim at 5.5 m/s produced the highest severity, with peak linear accelerations exceeding 700 g for some suspension-based helmets, followed by rear impacts. In the single helmet model evaluated, MIPS reduced peak rotational acceleration. Finally, the influence of the surface type on peak head kinematics was borderline significant, with P80 papers producing larger peak kinematics.

**Conclusion:**

Helmet design has a key role in protection against trip and fall impacts, with foam-based helmets providing added benefits. These findings highlight the need for improvements in helmet safety standards and helmet designs to better prevent work-related brain injuries.

## Introduction

Incidents in workplace can lead to traumatic brain injuries. Despite the mandate for head protection under the Personal Protective Equipment Regulations 1992 in the UK and strict enforcement, there were 348,453 work-related traumatic brain injury (Wr-TBI) cases admitted to hospitals in 2019-20 [1]. Similarly, in the United States, where 2 to 3 million TBIs occur annually, an estimated 18 % are work-related [2, 3]. These Wr-TBIs impose a considerable economic burden. In the UK alone, the total cost of workplace injuries was estimated at £ 7.7 billion in 2021-22 [4].

Industrial safety helmets are used to protect the head in workplaces. They were first mass-produced in the late 1800s, with a steel plate riveted inside. Industrial helmets are designed to pass current standards, EN 397 in Europe and ANSI Z89.1-2014 in the US [5, 6]. According to their test methods, rounded and pointed strikers are dropped on the crown of the helmet to test its protection against penetration and shock absorption from falling objects.

A recent analysis of Swedish Work Environment Authority and German Social Accident insurance data shows that only 17 % of TBIs are caused by falling objects, in contrast to trips and falls, which cause 50–70 % of TBIs [7–9]. Another study shows that in industrial accidents, the impact location is often in the front region of the helmet (up to 46 %), followed by the crown (up to 28 %), the rear (up to 20 %), and finally the sides (up to 16 %) [10, 11]. Additionally, most accidents occur when people are looking partially down or ahead, which can reduce the likelihood of direct crown impacts [12]. However, crown impacts are still possible, especially when the head is in a relatively neutral posture. While industrial helmets are effective in mitigating head injuries from falling objects on the crown [12–14], they provide poorer protection against impacts to the side, front, and rear [13, 15]. Moreover, many industrial helmets lack a chin strap or secure retention system, increasing the likelihood of helmet detachment during trip or fall scenarios. Current standards like EN 397 and ANSI Z89.1-2014 do not evaluate helmet retention performance during dynamic impacts, unlike standards for bicycle or climbing helmets. This further highlights the limitations of current standards.

In recent years, there has been growing interest in improving impact protection for those working at height. This has led to the use of expanded polystyrene (EPS) foam and optional chin straps. One example is the climbing-style helmet, which has gained popularity for its brimless design and integrated chin strap. Among industrial helmets incorporating EPS foam, both the amount and coverage vary by model. Some retain the traditional suspension system alongside the foam, while others replace the suspension entirely with a foam pad. Rotational reducing technologies, such as the Multidirectional Impact Protection System (MIPS), which has been shown to reduce head rotation and the risk of TBI in bicycle helmets [16], are also being adopted. These innovations have expanded the design landscape beyond conventional industrial helmets.

There are, however, minimal data to support the effectiveness of these new safety helmet designs in trips and falls. In a recent study, a fall-specific impact testing framework was proposed to benchmark helmet performance under falls from greater heights, representing more severe impact scenarios [17]. While this work provides valuable benchmark data, test methods reflecting typical workplace trip and fall conditions remain limited. Therefore, there is a need for test methods that can evaluate helmets under impact conditions representing trips and falls. In the absence of real-world data, a recent work simulated workplace trip and fall scenarios to determine head impact conditions [18]. The analysis revealed trends consistent with real-world observations, predicting that 55–68 % of head impacts occur to the front of the head, which is similar to real-world data indicating that frontal impacts occur in 38-46 % of cases and represent the highest proportion overall [12]. Based on these simulations, five representative impact conditions were identified, reflecting common trips and falls. These conditions are defined by the impact speed, angle, and location. A critical question remains regarding the level of protection that current industrial helmets offer under these conditions, assuming the helmet remains properly positioned and retained during impact.

This study evaluates the performance of different industrial helmets, including those with suspension system and EPS liner, under impact conditions representative of trips and falls. Helmets were exposed to impacts at different locations to assess how impact location influences protective performance and to identify areas for design improvement. Since surface friction can vary in real-world falls and trips, impacts were conducted on surfaces with different coefficients of friction to assess their effect on helmet response. Additionally, we evaluated two helmets of the same model, one equipped with MIPS and the other without, to provide an initial assessment of MIPS’ effect on protection during simulated trips and falls. This study highlights areas for helmet design improvement and demonstrates how simulated trip and fall impacts can distinguish performance among different industrial helmets.

## Methods

### Industrial Helmets

A list of over 30 industrial helmets available in the UK market was compiled using selection criteria that included a range of price points, best-sellers from major retailers, well-known manufacturers, and various helmet types (suspension-based and foam-based) as well as different brim styles (short and long peaks). One challenge in selecting these helmets was the limited availability of individual consumer reviews, as most industrial helmets are purchased by companies, leaving few reviews online. Additionally, the list needed to include helmets that meet different safety standards.

**Fig. 1:**
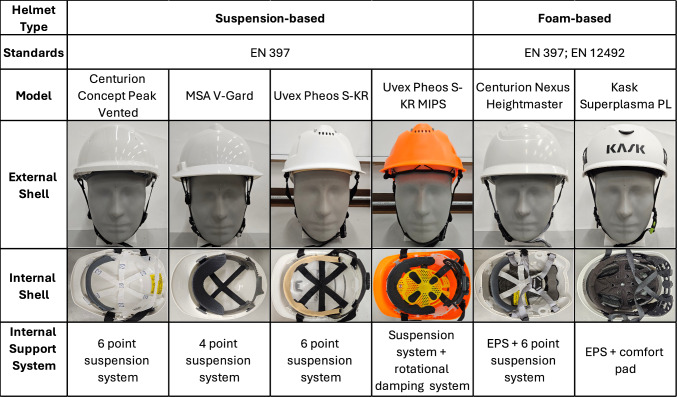
The industrial helmets included in the study, their external and internal shell designs, and the European safety helmet standards they meet

Six different helmets, representing a range of industrial helmet types, were selected for testing (Figure 1). These included traditional helmets with suspension systems which meet the EN 397 standard [6] and the increasingly popular climbing-style helmets that comply with both EN 397 and EN 12492 standards [19]. The selection also included helmets with and without EPS foam, and one equipped with a dedicated rotation reducing system, MIPS. The helmets varied in price from £ 4.80 to £ 49, reflecting a broad spectrum of prices and designs. Some of these helmets do not come with chin straps by default; chin straps specifically designed by the manufacturer for these helmets were purchased and fitted to ensure they remained securely on the headform throughout the tests.

### Impact Conditions Representing Trips and Falls

**Fig. 2:**
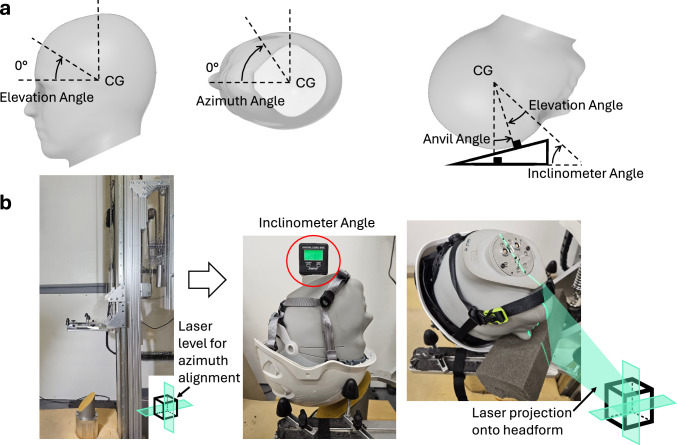
The positioning of the helmeted headform. **A** The definition of the angles used to position the headform. **B** Helmet fitting and positioning setup showing the inclinometer used to set the elevation angle and the laser level positioned beside the drop tower to define the azimuth alignment plane by projecting a horizontal beam along the intended impact direction. The right image illustrates the azimuth alignment procedure, while the schematic inset shows the orientation of the laser plane relative to the laboratory coordinate system and its alignment with the headform

Five test conditions were selected, based on recent simulations of trips, forward falls, and backward falls [18]. These test conditions are defined by the impact speed, impact angle, and impact location defined by the elevation and azimuth angles (Figure 2A). The distribution of these parameters based on 1692 simulations were used to define the conditions. The 75^th^ percentile value of the impact speed, the mean value of the impact angle, and the 50^th^ percentile value of the elevation and azimuth angles were used to guide the selection of the impact conditions [18].

Pilot testing was conducted to select five different impact conditions, representing trips and falls. Testing revealed that impacts closer to the rim of the helmet significantly increased linear head kinematics. Hence, a forward fall scenario using the 25^th^ percentile value of the elevation angle from the simulated falls was included in the impact conditions [18]. Testing further revealed that an impact speed of 5.5 m/s produced very large peak linear accelerations (PLA) in some helmets. Hence, the impact speed was limited to 5.5 m/s for forward and backward falls. This enables comparisons of helmet safety performance between front and rear impacts under similar conditions. The five impact conditions used in this study are detailed in Table 1.

**Table 1:** Impact conditions selected for experimental testing based on recent simulation results [18]. Snapshots depicting the headform’s orientation just before impact for each of the five conditions are seen in Figure 3 at the 0 ms timestamp

Impact conditions	Trip	Forward fall 45°	Forward fall	Forward fall rim	Rear fall
Impact speed (m/s)	3.9	3.8	5.5	5.5	5.5
Impact angle^1^	75	45	75	75	75
Elevation angle	35	45	40	25	40
Azimuth angle	45	0	0	0	180
Inclinometer angle^2^	40	0	35	50	35

^1^All angles are in degrees

^2^Equation 1

### Experimental Setup

The helmets were tested under impact conditions listed in Table 1, using the helmet test rig at the Human Experience, Analysis and Design (HEAD) Lab, Imperial College London. The rig consists of a guided free-fall system, where a helmeted headform is positioned onto a U-shaped testing platform that drops vertically along precision guide rails (Figure 2B). The U-shaped carriage guides the helmeted headform during the free fall. Upon the initiation of contact between helmet and anvil, the helmeted headform detaches from the carriage. The carriage continues its fall until stopped by a thick layer of foam. This system has been used in prior studies to evaluate helmet performance [16, 20–22].

We used the CEN headform manufactured by Cellbond (Cellbond Composites Ltd, Huntingdon, United Kingdom) in 2024 (Figure 2). This is a new headform developed by the Working Group 11 of the European Standardisation head protection Committee (CEN/TC158/WG11) and recently published in the EN 17950 standard [23]. The physical properties of this headform are detailed in Table 2. All properties fall within the corridors defined by the standard, except MoI xx, which is 4.5% above the upper limit defined by the standard.

The headform was instrumented with a DTS 6DX PRO sensor package (Diversified Technical Systems, Seal Beach, CA, USA) managed by a wireless SLICE Nano data logger system. This system recorded linear accelerations and rotational velocities along the x, y, and z axes using tri-axial accelerometers with a range of ± 2000 g and tri-axial angular rate sensors with a range of ± 8000 deg/s.

Data were collected 0.5 seconds before and after each impact at a sampling frequency of 20 kHz. Linear acceleration and rotational velocity signals were filtered using cutoff frequencies of CFC 1000 and CFC 180, respectively, following ISO 6487 standard [24]. The filtering was performed using a fourth-order Butterworth phaseless digital filter function, implemented in accordance with SAE J211-1 guidelines [25]. These filtered signals were used to calculate resultant values and to derive rotational acceleration by differentiating the rotational velocity. Based on prior evaluation of N-point moving averages for smoothing, a 1-point moving average (no smoothing) was determined to be sufficient for differentiation [16]. Therefore, the 1-point method was applied for differentiation.

**Table 2:** Physical properties of 2024 version of Cellbond-CEN headform

Circumference [mm]	Mass [kg]	MoI^1^ xx [kg.cm^2^]	MoI yy [kg.cm^2^]	MoI zz [kg.cm^2^]	CoF^2^
570^3^	4.26^3^	218.9 ± 1.2^3^	238.9 ± 0.9^3^	158.2 ± 1.0^3^	0.33 ± 0.01^4^

^1^Moment of inertia

^2^Coefficient of friction

^3^Measurements performed by Cellbond.

^4^Tested in the HEAD Lab, Imperial College London on 21^st^ June 2024, prior to helmet tests. COF was measured between a PES (polyester) strap and headform surface using the method explained in [25] and [22].

The helmet was secured to the headform according to the manufacturer’s instructions. A 10-mm-diameter rod was placed between the chinstrap and chin, and the chinstrap was tightened. Then, the rod was removed to ensure a 10 mm gap between the chin and the strap. A 2-cm-thick layer of low-density foam, measuring 15 cm by 10 cm, was applied to the front of the headform to protect the unhelmeted areas from damage during impact.

The helmeted headform, along with a small foam support block, was then placed on the U-shaped testing platform and precisely orientated using a digital inclinometer (accuracy of ± 1 °) and, where necessary, a laser level to achieve the required position. The inclinometer was used to set the elevation angle, measuring from the base of the headform (Figure 2). The inclinometer angle was calculated as follows (Table 1):

1 $$Inclinometer \, angle\, = \,{9}0^\circ {-}elevation \, angle{-}anvil \, angle.$$

After setting the elevation angle of the headform, a laser level was used to set the azimuth angle. The laser level was positioned at the desired azimuth angle to define the horizontal direction of impact around the headform (Figure 2B) [18]. The headform was then rotated until the posterior-anterior reference line marked on its base aligned with the laser level. The mass of the additional foam was ~ 0.03 kg, which is negligible compared with the 4.27 kg mass of the headform.

The headform was then dropped onto either a 45 ° or 15 ° anvil to simulate impact angles of 45 ° and 75 °. The anvil was covered with two different surfaces: the 80-grit abrasive paper (P80) and roof shingle (Figure 2). Hard hats are primarily used in construction, mining, and industrial environments, which involve a wide range of surface conditions. In this study, we used roof shingles to represent fine concrete and asphalt surfaces, as previous work has shown that roof shingles have similar surface characteristics to these materials and produce similar head kinematics [26]. The same study demonstrated that the coefficient of friction (CoF) between a helmet and P80 abrasive paper is higher than the CoFs between a helmet and either concrete or asphalt. Therefore, we used P80 abrasive paper to simulate rougher surfaces, such as unfinished or improvised flooring that may be encountered in construction and mining environments, which are expected to have higher friction than smooth, trowel-finished concrete. While the P80 surface does not precisely replicate any specific rough flooring type, it serves as a reasonable approximation to explore head impact behaviour under higher-friction surface conditions. Each test was repeated three times using a new helmet sample. In total, 30 helmets were tested across five impact conditions and two surface types for each of the six helmet models.

### Brain Injury Metrics

Linear acceleration and rotational velocity vectors measured by the sensors were used to derive kinematic injury metrics commonly used to predict brain injuries. These metrics include PLA, peak rotational acceleration (PRA), peak rotational velocity (PRV), and the brain injury criterion (BrIC).

2 $$BrIC = \sqrt {\left( {\frac{{\max \left( {\left| {\omega_{x} \left( t \right)} \right|} \right)}}{{\omega_{xC} }}} \right)^{2} + \left( {\frac{{\max \left( {\left| {\omega_{y} \left( t \right)} \right|} \right)}}{{\omega_{yC} }}} \right)^{2} + \left( {\frac{{\max \left( {\left| {\omega_{z} \left( t \right)} \right|} \right)}}{{\omega_{zC} }}} \right)^{2} },$$

where ${\omega }_{x}\left(t\right), {\omega }_{y}\left(t\right),$ and ${\omega }_{z}\left(t\right)$ are the components of the head rotational velocity. The critical rotational velocities are ${\omega }_{xC}$ = 66.25 rad/s, ${\omega }_{yC}$ = 56.45 rad/s, and ${\omega }_{zC}$ = 42.87 rad/s [27].

### Data Analysis

High PLAs were measured in certain scenarios, e.g. Forward fall rim impacts. When PLA exceeded 250 g, we excluded the helmet from further analysis. This 250 g threshold corresponds to a 67 % risk of skull fractures based on a risk function developed by combining post-mortem human subject (PMHS) tests, finite element modelling, and HIII drop tests [28]. In addition, 250 g corresponds to a 69 % risk of AIS4 + head injuries, including skull fractures, subarachnoid haemorrhage, and contusions, based on a study involving 30 elderly vulnerable road users [29]. It also aligns with the pass/fail limit set by several helmet standard [5, 6, 19]. Hence, we chose 250 g as a conservative and biomechanically justified exclusion criteria.

For each helmet, the mean value, standard deviation, and coefficient of variation (CV) were calculated across all repeats for each injury metric. The CV was used to assess helmet test variability. We assessed the effects of impact location and surface condition on helmet performance metrics (PLA, PRA, PRV, and BrIC) using linear mixed-effects modelling in R (version 4.3.3). This modelling approach was chosen because it appropriately handles unbalanced datasets and missing data, which in this case arose when certain trials were omitted after exceeding the 250 g PLA limit. We developed separate models for each metric, with surface (P80, roof shingle), location (Forward fall 45 °, Trip, Forward fall, Forward fall rim, Rear fall), and their interaction as fixed effects. Helmet model was included as a random intercept to account for repeated measures within helmets. Model assumptions were checked by assessing residual skewness and normality. Right-skewed variables were log-transformed based on their distribution and improvements in model fit (Akaike Information Criterion).

Significance of fixed effects was assessed using likelihood ratio tests comparing models with and without each term. For any significant fixed effect, post hoc pairwise comparisons between factor levels were performed using estimated marginal means with Bonferroni correction for multiple comparisons. All analyses were conducted in R using the lme4 (version 1.1-37), lmerTest (version 3.1-3), and emmeans (version 1.11.2-8) packages. Mann–Whitney U test was performed to compare the performance of the helmet with and without MIPS.

## Results

### Overall Head Kinematics

**Fig. 3:**
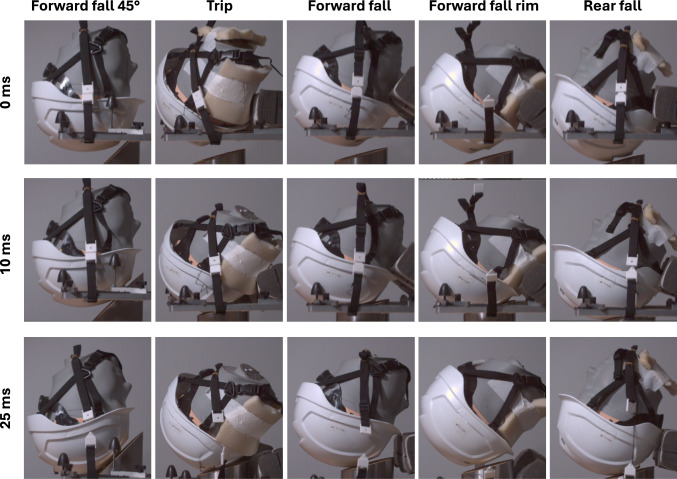
Snapshots from high-speed footage capture the sequence of an industrial helmet’s response under five different impact conditions, from just before contact with the anvil (0 ms) to full contact with the anvil (10 ms), and finally, the moment of separation from the anvil (25 ms)

Figure 3 presents snapshots from high-speed video footage capturing a helmet under five distinct impact conditions. The frame at 10 ms shows the moment of full contact, where the helmet is fully compressed. By 25 ms, the helmet has fully separated from the anvil and returned to its original shape, with head accelerations dropping to zero across all conditions (Appendix 1).

For impacts on the 15 ° anvil, the headform predominantly rebounds vertically off the anvil, while for the impact on the 45 ° anvil (Forward fall 45 °), it rebounds to the left. These directions are expected based on the direction of the force normal to the anvil.

### Inconsistent Results between Repeats for Suspension-based Helmets

**Fig. 4:**
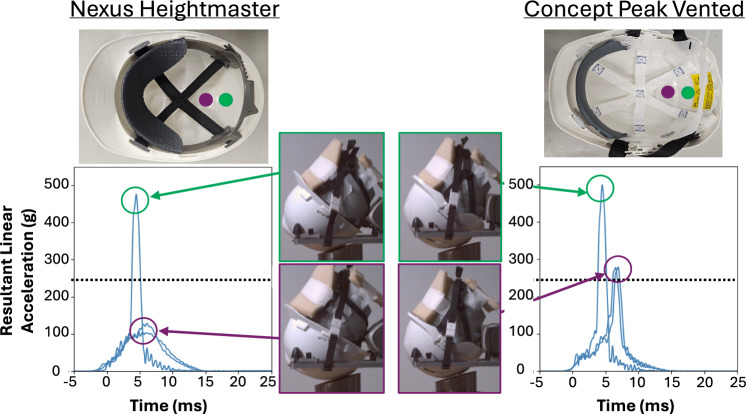
Drastic variation in resultant linear acceleration due to minor differences in impact location within Rear fall scenario, despite head orientation being maintained within the ± 1 ° tolerance specified in the experimental methods. Impact locations are highlighted in green and purple on each helmet, corresponding to the circled peaks in the resultant linear acceleration graphs

The resultant linear acceleration varied significantly between repeats in some suspension-based helmets. Notably, one helmet (Nexus Heightmaster) in the Forward fall condition and two helmets (Concept Peak Vented and V-Gard) in the Rear fall condition exhibited this inconsistency. Figure 4 shows two suspension-based helmets tested under the Rear fall scenario on a P80 surface. The PLA in this scenario ranged from 113.5 g to 484.9 g V-Gard and 281.3 g to 508.6 g in Concept Peak Vented. The time history of resultant linear acceleration shows extremely elevated PLA, indicating "bottoming out". There was no visible damage such as broken straps or cracked external shells in the helmets. High-speed footage revealed variations in impact location between repeats, despite head orientation being within the pre-impact tolerance. Impacts that were slightly inclined towards the rear of the helmet consistently recorded significantly elevated PLA, as highlighted in green in Figure 4.

### Only a Foam-Based Helmet Reduced PLA Below 250 g in all Impacts

**Fig. 5:**
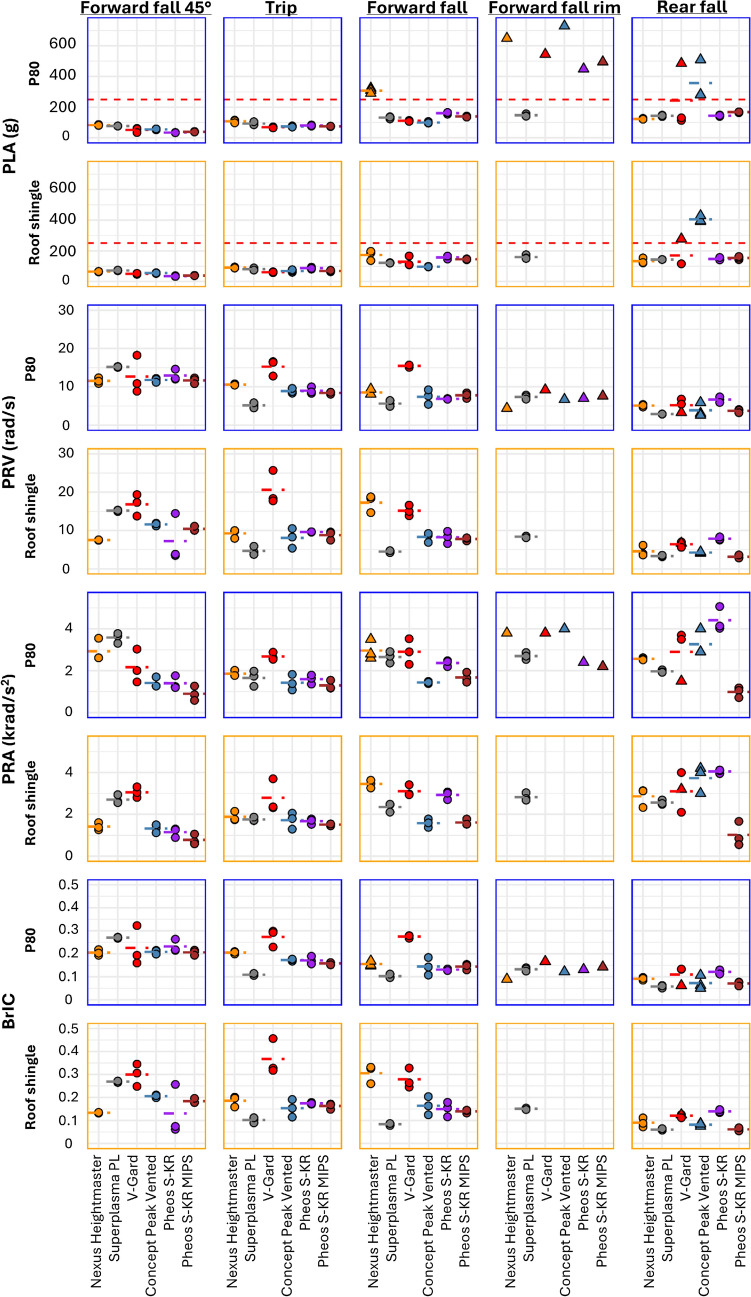
PLA, PRV, PRA, and BrIC values for all helmets across impact and surface conditions (three repetitions per test). The red dashed line in the PLA panels marks the 250 g right-censoring threshold, with data points above this line representing impacts that reached this limit. Missing data points indicate instances where at least one repetition exceeded the 250 g PLA limit and the test was not repeated. Dashed horizontal lines represent the mean value for each helmet when all three repetitions were available. Tests with PLA > 250 g are shown as triangles, and their corresponding PRV, PRA, and BrIC values are also plotted as triangles

Figure 5 shows that PLA responses differed significantly by helmet type and impact condition. Under more severe impact conditions, such as higher impact speeds and impacts closer to the helmet rim, fewer helmet models achieved PLA below 250 g. All six models met this limit in the Forward fall 45 ° and Trip conditions, while only five did so in Forward fall. This number further decreased to three models in Rear fall and just one model in Forward fall rim. For helmets exceeding the 250 g limit, PLA values ranged from 277.8 g to 728.8 g depending on the helmet type and impact condition. The only helmet that reduced PLA below 250 g across all impact conditions and surface types was a foam-based model (Superplasma PL), with a highest average PLA of 159.4 g. PRA, PRV, and BrIC values for each impact location, surface condition, and helmet model are presented in Figure 5**.**

### Variations in Kinematic Metrics

Among the helmets with PLA < 250 g, only 2 out of 45 (4.4 %) tested helmet-impact locations recorded a CV of PLA exceeding 20 % (Appendix 2, Table 4). We observed larger variations in PRA and PRV, particularly in suspension-based helmets. Specifically, the CV of PRV exceeded 20 % for 6 (13.3 %) helmet-impact location combinations and CV of PRA exceeded 20 % for 10 (22.2 %) helmet-impact location combinations, with one extreme case exceeding 80 %.

### Peak Head Kinematics Influenced by Impact Conditions, With Minor Effects of Surface Condition

**Fig. 6:**
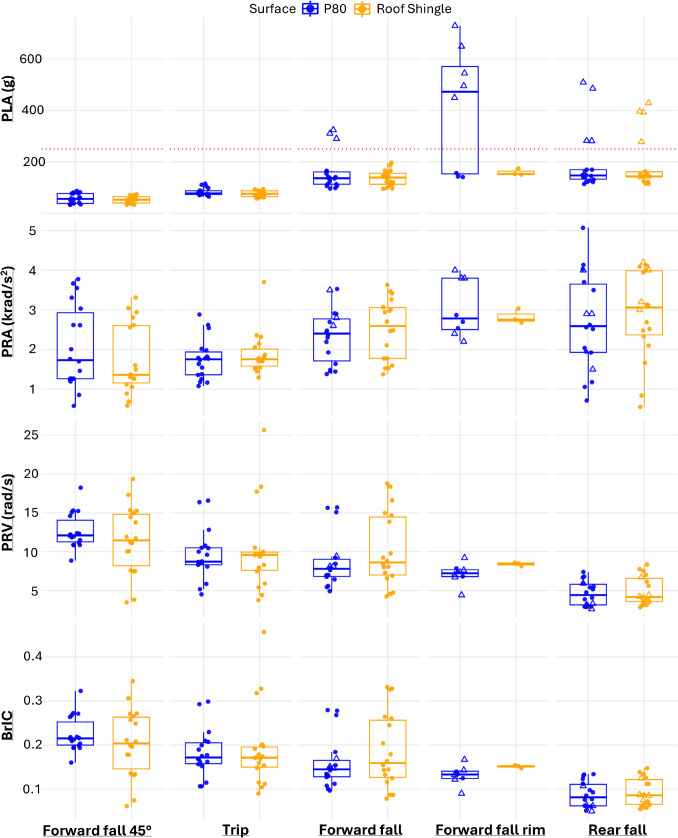
Influence of surface condition (P80 and roof shingle) across all impact locations and helmet models. The dashed red line in the PLA panel indicates the 250 g threshold used for censoring. Unfilled triangles (△) denote impacts exceeding this threshold (PLA > 250 g), and the same trials are marked correspondingly in the PRA, PRV, and BrIC panels

Peak kinematics for different impact and surface conditions are shown in Figure 6. PLA, PRA, and PRV were analysed on the log-transformed scale to reduce skewness, while BrIC was analysed untransformed. Mixed-effects models showed that impact location had a significant effect on all four kinematic metrics (PLA: χ^2^(8) = 197.5, *p* < 0.001; PRA: χ^2^(8) = 52.3, *p* < 0.001; PRV: χ^2^(8) = 78.6, *p* < 0.001; BrIC: χ^2^(8) = 68.2, *p* < 0.001). A small but significant effect of surface condition was observed for PLA (χ^2^(5) = 11.9, *p* = 0.036), PRA (χ^2^(5) = 10.8, *p* = 0.029), and PRV (χ^2^(5) = 9.84, *p* = 0.041), with higher values on P80 than on roof-shingle surfaces, but no effect for BrIC (χ^2^(5) = 4.37, *p* = 0.22). No significant surface–location interactions were detected for any metric (all *p* > 0.07).

The mean PLA across all helmets and surfaces was highest for Forward fall rim (344 ± 231 g), followed by Rear fall (194 ± 111 g) and Forward fall (148 ± 56 g), with lower values for Trip (79.5 ± 13.7 g) and Forward fall 45 ° (54.5 ± 16.5 g) impacts. Post hoc tests (Bonferroni-adjusted) showed that Forward fall rim, Forward fall, and Rear fall produced significantly higher PLA than Trip and Forward fall 45 ° impacts (*p* < 0.001).

For PRA, the mean was highest for Forward fall rim (2.98 ± 0.61 krad/s^2^), followed by Rear fall (2.79 ± 1.15 krad/s^2^) and Forward fall (2.42 ± 0.72 krad/s^2^). Lower values were observed for Trip (1.82 ± 0.52 krad/s^2^) and Forward fall 45 ° (1.90 ± 0.97 krad/s^2^). Post hoc comparisons indicated higher PRA for Forward fall rim, Forward fall, and Rear falls than for Trip and Forward fall 45 ° impacts (p < 0.001).

The mean PRV was greatest for Forward fall 45 ° (12.0 ± 3.48 rad/s), followed by Trip (9.86 ± 4.40 rad/s) and Forward fall (9.41 ± 4.14 rad/s). Lower rotational velocities were found for Forward fall rim (7.47 ± 1.28 rad/s) and Rear fall (4.75 ± 1.69 rad/s). Post hoc tests showed that Forward fall 45 ° and Trip impacts produced significantly higher PRV than Rear falls (*p* < 0.001).

The mean BrIC was highest for Forward fall 45 ° (0.214 ± 0.061), Trip (0.187 ± 0.074), and Forward fall (0.173 ± 0.073), with lower values for Forward fall rim (0.137 ± 0.021) and Rear fall (0.090 ± 0.030). Post hoc tests revealed that Forward fall 45 ° and Trip impacts produced significantly higher BrIC values than Rear falls (p < 0.001).

### Different Directions of Rotation

When examining the direction of rotation, we found differing patterns across impact and surface conditions. On the roof-shingle surface, the headform generally rotated towards the anvil after bouncing off in Trip, Forward fall, Forward fall rim, and Rear fall impact conditions. In contrast, the Forward fall 45 ° impact condition typically produced rotation away from the anvil. On the P80 surface, Trip, Forward fall, and Forward fall rim impacts also tended to result in rotation towards the anvil, whereas Forward fall 45 ° produced rotation away from the anvil. The Rear fall impact condition on P80, however, showed approximately equal numbers of rotations in both directions. These general trends, however, were not consistent. Variation was seen across helmet types and even between repeated trials, particularly on the roof-shingle surface.

**Fig. 7:**
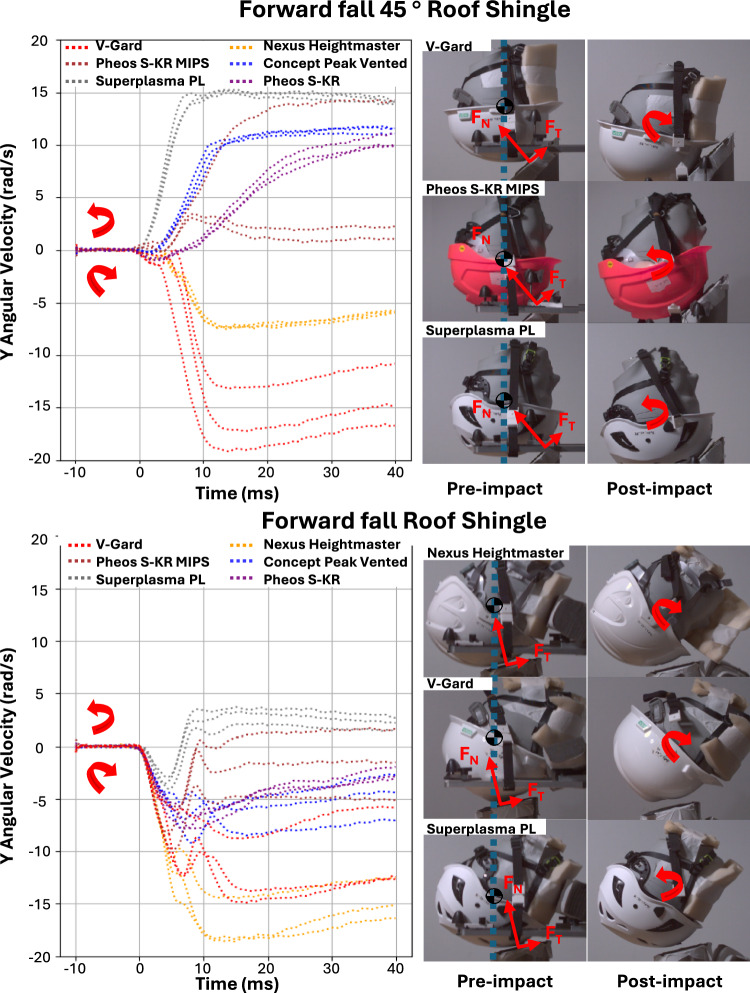
Variation in the direction of rotation under Forward fall 45 ° and Forward fall conditions, shown through the Y-axis component of the velocity-time graph. Negative angular velocity indicates rotation towards the anvil, while positive angular velocity indicates rotation away from it. High-speed video footage validates the rotational motion, illustrating the headform’s movement pre- and post-impact

To better clarify this point, in Figure 7, we have shown the Y-axis component of the rotational velocity for Forward fall 45 ° and Forward fall impacts across all helmet types on the roof-shingle surface. In the velocity-time graph, a negative Y-angular velocity indicates rotation towards the anvil, while a positive value indicates rotation away from it. This figure shows that in Forward fall 45 °, 2 helmet types rotate towards the anvil, while 4 rotate away. Under Forward fall conditions, only 1 helmet type rotates away from the anvil, while the remaining helmets rotate towards it.

### The Effects of MIPS between Impact Conditions in a Single Helmet

To evaluate whether MIPS provides a statistically significant protective advantage, Mann–Whitney U tests were conducted comparing the Pheos S-KR MIPS helmet to its non-MIPS counterpart. A total of 48 tests were analysed, consisting of three repeats for each combination of four impact scenarios (Trip, Forward fall 45 °, Forward fall, Rear fall), two surfaces (P80 and roof shingles), and helmets with and without MIPS. Forward fall rim was excluded, resulting in 24 tests per helmet type. The results in Table 3 show a significant 46 % reduction in PRA for the MIPS-equipped helmet. Although the helmet with MIPS demonstrated a reduction in average PLA by 2.1 %, PRV by 9.8 %, and BrIC by 9.8 %, these reductions were not statistically significant.

**Table 3:** A comparison on the effect of MIPS across all five impact conditions and both surface types (P80 and roof shingle) for one helmet model with MIPS (Pheos S-KR MIPS) and without MIPS (Pheos S-KR)

Metric	With MIPS (*n* = 24 tests)	Without MIPS (*n* = 24 tests)	% Difference with MIPS	Mann Whitney U Test
PLA (g)	103.3 ± 50.9	105.5 ± 51.4	−2.1	U = 260, *p* = 0.877
PRA (krad/s^2^)	1.2 ± 0.4	2.4 ± 1.2	−46.0	U = 206, *p *< 0.0001
PRV (rad/s)	7.7 ± 2.9	8.6 ± 2.8	−9.8	U = 243, *p* = 0.705
BrIC	0.141 ± 0.049	0.157 ± 0.049	−9.8	U = 238, *p* = 0.602

Head kinematics were also compared between the Pheos S-KR MIPS and without MIPS helmet in individual impact conditions. MIPS significantly reduced the average PLA by 13.7 % in Trip (*p* = 0.002) and by 10.6 % in Forward fall (*p* = 0.004). In contrast, PLA increased by 14.2 % in Forward fall 45 ° (*p* = 0.004) and by 9.9 % in Rear fall (*p* = 0.041) when using MIPS. MIPS significantly reduced the average PRA in Forward fall 45 ° by 34.2 % (*p* < 0.05), Forward fall by 38.0 % (*p* < 0.01), and Rear fall by 71.3 % (*p* < 0.01). Additionally, significant reductions in PRV and BrIC were observed with MIPS under Rear fall conditions, showing decreases of 52.6 % (*p* < 0.01) and 49.3 % (*p* < 0.01), respectively.

## Discussion

This study shows that the safety performance of industrial helmets under representative trip and fall conditions varies significantly depending on the helmet design and impact conditions. We evaluated four suspension-based and two foam-based industrial helmets under five impact conditions derived from a previous study that has simulated workplace trips and falls [18]. The maximum impact speed was 5.5 m/s corresponding to falls from a height of approximately 2 to 2.5 m [30]. This fall height is consistent with findings from a study of 1,651 patients who sustained work-related fall injuries, which identified 2.2 m as the median fall height [31]. We found that although both foam-based and suspension-based helmets provide protection against some falls and trips at different locations and speeds, only one foam-based helmet reduced PLA below 250 g across all impact conditions. Since trips and falls account for 50–70 % of TBIs in the workplace [7–9], our results highlight an important gap, and an opportunity for design improvement, in the protective performance of industrial helmets.

Recent fall-specific helmet-impact testing has examined rear impacts at speeds comparable to those used in the present study [17]. We observed higher peak linear accelerations in some rear impacts, likely due to impacts occurring closer to the helmet rim, outside the primary support region. More generally, we found that a slight shift in impact location led to significantly different peak head kinematics for suspension-based helmets. In contrast, helmets with full EPS coverage showed less sensitivity to the impact location. Additionally, moving the impact closer to the helmet rim, from Forward fall to Forward fall rim, resulted in PLA well above 250 g, particularly in suspension-based helmets. In one helmet, PLA reached 728.8 g, exposing a critical vulnerability at the rim. When comparing front (Forward fall) and rear (Rear fall) impacts under the same conditions, there were no considerable differences in PLA or PRA for foam-based helmets, but there were notable changes for suspension-based helmets, indicating that impact location can greatly influence performance based on helmet design. However, the foam-based helmets did not offer uniformly superior linear protection, in contrast to the finding of a previous study [32]. In our study, one foam-based helmet exceeded the 250 g PLA limit under the frontal impact close to the rim (Forward fall rim). Our results highlight the need for a more comprehensive evaluation of industrial helmets beyond the current EN 397 and EN 12492 standards, which focus primarily on crown and side impacts from falling objects. These findings suggest the need to include a broader range of impact locations, angles, and speeds, and advocate for more rigorous testing conditions to better assess industrial helmets.

Although high-speed footage showed visible differences in helmet slip and rotation between the P80 and Roof-Shingle surfaces, these did not result in statistically significant differences across any injury metrics. Specifically, variation in rotational motion, including negative and positive rotations, occurs as some helmets slip on the anvil due to a low CoF and therefore F_T_. However, the effects of surface conditions on injury metrics are small under the simulated falls and trip. Instead, our results underscore the importance of testing helmets at various impact locations to address linear protection effectively. This focus is crucial, given the poor performance of most helmets in reducing linear acceleration at an impact speed of 5.5 m/s. This impact speed corresponds to a fall height of approximately 2 to 2.5 m, which is a common fall height, even for individuals not specifically working at height, such as falling from a ladder [33].

We observed that the distance between F_N_ vector and CoG of the headform plays an important role in determining the direction of headform’s rotation. When F_N_ was applied closer to the CoG, F_T_ had a greater rotational effect, often causing the headform to rotate away from the anvil (Figure 7). In contrast, when F_N_ was applied further from the CoG, the headform tended to rotate towards the anvil. The suspension-based helmets, which tend to have a taller design, increased the F_N_–CoG distance, thus allowing F_N_ to dominate the rotational response. These findings align with prior studies that reported similar relationship between the impact point–CoG distance and headform rotation [34, 35]. Here, we report this relationship for industrial helmets under simulated fall and trip conditions, highlighting the influence of helmet design.

MIPS has often been demonstrated to be effective in reducing rotational motion under bicycle helmet impacts [16, 36, 37]. This study is the first to show that MIPS remained effective in reducing rotational motion in an industrial helmet. The reductions were more pronounced in rear impacts, where PRA, PRV, and BrIC were all reduced by the helmet equipped with MIPS compared with its non-MIPS version. However, the reductions seen in the specific conditions of this study are not as pronounced as those seen in bicycle helmet tests. This can be because MIPS is more effective under impact conditions that produce large head rotations due to a dominant tangential force component (F_T_), e.g. for impacts on a 45 ° anvil at higher speeds.

The rotational kinematics measured in our experiments were relatively low, which we attribute to the dominance of the normal force component (F_N_) and the small distance between the F_N_ vector and the headform’s CoG. While falls from height typically produce a primarily vertical (normal) impact, real-world workplace scenarios also include same-level trips and side impacts, where the fall dynamics may introduce larger F_T_ or F_N_–CoG distance. These scenarios can result in greater head rotation. Future research should explore test conditions that better capture these variations, in order to more fully represent the range of real-world events that contribute to work-related TBIs.

This study did not address impacts from falling objects. Although such scenarios often result in less severe injuries in real-world situations, they still account for 17 % of reported wr-TBIs [9]. Additionally, studies have shown that objects striking slightly off-centre from the crown can produce significant rotational motions [38]. Availability of in-depth real-world data can provide invaluable insights into such mechanisms of wr-TBI and guide the development of better test methods.

Helmet retention system is helmet retention. In many workplaces, helmets are worn without chin straps, making them more likely to fall off during a trip or fall. This greatly reduces their ability to protect the head, especially for side, front, or rear impacts. While our tests assumed helmets stayed properly positioned, this may not reflect real-world use. Future standards should include tests for helmet stability during impacts, and greater emphasis should be placed on encouraging or requiring chin strap use on-site.

The conclusions of this study are constrained by other limitations. First, we used six different helmets only, which may not allow us to generalise the findings. Industrial helmets have a wide range of design features, with variations including external elements like humps or EPS of different thicknesses. Nonetheless, the selected impact conditions were able to distinguish between the performance of the helmets. The conclusions related to the efficacy of MIPS are limited to the single helmet which has MIPS and no-MIPS versions. Additionally, the impacts in this study were limited to a small surface area of the anvil rather than a continuous floor. This could especially affect helmets with unique external design features, where parts of the helmet or the rim might contact and clip the edge of the anvil, leading to inconsistent injury metrics. Another limitation of this study is the observed variation in headform position between repetitions during free-fall tests for some helmets. Despite efforts to maintain positioning within the required tolerances through angle measurements, high-speed video revealed inconsistencies that affected rotational motion. Additionally, for the Trip and Forward Fall Rim impact conditions, a foam block was loosely attached to the front of the headform to protect unhelmeted areas from damage upon rebound. While the foam did not restrict helmet movement during impacts, some minor resistance may have occurred, potentially influencing headform kinematics in these conditions. Finally, the Cellbond-CEN headform’s moment of inertia about the x-axis was 4.5 % above the upper bound defined in EN17950. While this can influence the head kinematics, particularly rotational kinematics, it should have minimal effects on the results derived from comparisons, such as the effects of surface condition and impact location, because the same headform has been used across all tests.

In summary, our evaluation of six commercially available industrial helmets under the new impact conditions shows the following:

- The selected impact scenarios, representing trips and falls, effectively differentiate helmets based on their head injury protection performance.
- Helmets with full EPS coverage have the potential to reduce linear acceleration across all impact conditions.
- Suspension-based helmets can perform very differently with slight changes in impact location.
- Helmet rim can substantially increase the risk of head injury, with PLA reaching values over 700 g in some suspension-based designs.
- The simulated impact conditions produce small rotational kinematics, which are predominantly influenced by the distance between the normal force and the CoG of the headform and the magnitude of the tangential force.

The results of this study, and future extensions of this research, hold promise for improving helmet designs and test methods to better prevent wr-TBI in real-world scenarios, including trips, falls, and falling objects.

## Figures and Tables

**Fig. 8:**
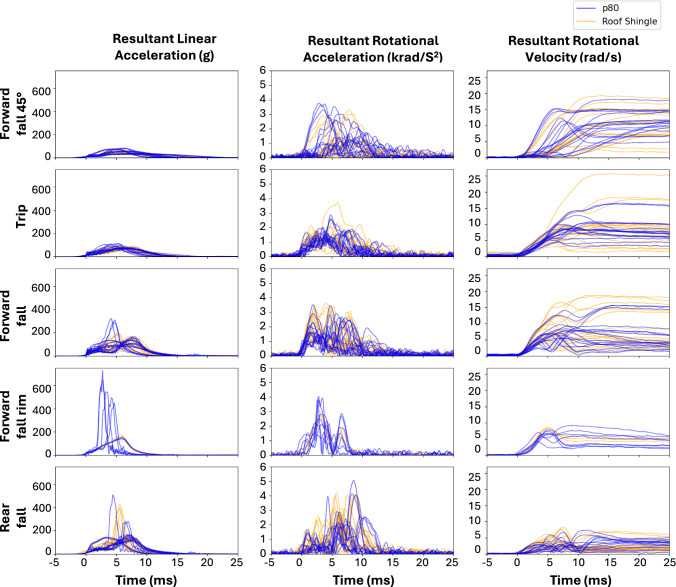
Time history of head kinematics, including resultant linear acceleration, rotational acceleration, and rotational velocity, for all helmet models and impact scenarios. Missing data indicate instances where at least one repetition exceeded the 250g PLA limit

## Appendix 1

See Fig. 8

## Appendix 2

See Table 4

**Table 4:** The kinematic results for all repetitions and helmet tests across impact scenarios: Forward fall 45 ° , Forward fall, Forward fall rim, Rear fall, and Trip

Test ID	Impact	Surface	PLA	PRV	PRA	BrIC
Centurion_Nexus_Heightmaster_01	Forward fall 45 °	p80	86.7	12.4	3.6	0.218
Centurion_Nexus_Heightmaster_02			80.8	10.9	2.6	0.194
Centurion_Nexus_Heightmaster_03			81.5	11.5	2.6	0.203
Mean			83.0	11.6	2.9	0.205
Standard Dev			3.2	0.7	0.5	0.012
Coefficient of Variance (%)			4	6	18	6
Centurion_Nexus_Heightmaster_01		Roof Shingle	62.5	7.4	1.3	0.132
Centurion_Nexus_Heightmaster_02			65.8	7.5	1.6	0.134
Centurion_Nexus_Heightmaster_03			65.5	7.6	1.4	0.135
Mean			64.6	7.5	1.4	0.134
Standard Dev			1.8	0.1	0.2	0.002
Coefficient of Variance (%)			3	1	12	1
Centurion_Nexus_Heightmaster_01	Forward fall	p80	323.7	8.1	2.6	0.147
Centurion_Nexus_Heightmaster_02			310.0	8.1	3.5	0.151
Centurion_Nexus_Heightmaster_03			289.9	9.4	2.8	0.169
Mean			307.9	8.5	3.0	0.155
Standard Dev			17.0	0.7	0.4	0.012
Coefficient of Variance (%)			6	9	15	8
Centurion_Nexus_Heightmaster_01		Roof Shingle	185.7	18.4	3.5	0.326
Centurion_Nexus_Heightmaster_02			196.6	14.7	3.3	0.260
Centurion_Nexus_Heightmaster_03			136.2	18.8	3.6	0.332
Mean			172.9	17.3	3.5	0.306
Standard Dev			32.2	2.3	0.2	0.040
Coefficient of Variance (%)			19	13	5	13
Centurion_Nexus_Heightmaster_01	Forward fall rim	p80	648.6	4.4	3.8	0.090
Centurion_Nexus_Heightmaster_02			Not done due to high PLA in test 1			
Centurion_Nexus_Heightmaster_03			Not done due to high PLA in test 1			
Mean			648.6	4.4	3.8	0.090
Standard Dev			0.0	0.0	0.0	0.000
Coefficient of Variance (%)			0	0	0	0
Centurion_Nexus_Heightmaster_01	Rear fall	p80	126.8	5.4	2.6	0.098
Centurion_Nexus_Heightmaster_02			121.7	4.7	2.6	0.085
Centurion_Nexus_Heightmaster_03			121.3	5.2	2.5	0.093
Mean			123.3	5.1	2.6	0.092
Standard Dev			3.1	0.3	0.1	0.006
Coefficient of Variance (%)			2	6	2	7
Centurion_Nexus_Heightmaster_01		Roof Shingle	153.4	6.1	3.1	0.112
Centurion_Nexus_Heightmaster_02			121.2	4.0	3.1	0.072
Centurion_Nexus_Heightmaster_03			123.9	3.6	2.3	0.088
Mean			132.8	4.6	2.9	0.091
Standard Dev			17.8	1.4	0.5	0.020
Coefficient of Variance (%)			13	30	16	22
Centurion_Nexus_Heightmaster_01	Trip	p80	115.2	10.8	1.8	0.210
Centurion_Nexus_Heightmaster_02			110.6	10.5	2.0	0.199
Centurion_Nexus_Heightmaster_03			97.7	10.4	1.8	0.207
Mean			107.8	10.6	1.9	0.206
Standard Dev			9.1	0.2	0.1	0.005
Coefficient of Variance (%)			8	2	8	3
Centurion_Nexus_Heightmaster_01		Roof Shingle	87.9	9.8	1.7	0.202
Centurion_Nexus_Heightmaster_02			94.3	10.0	1.8	0.197
Centurion_Nexus_Heightmaster_03			88.2	7.9	2.1	0.159
Mean			90.1	9.2	1.9	0.186
Standard Dev			3.6	1.1	0.2	0.023
Coefficient of Variance (%)			4	12	12	13
Centurion_Nexus_Heightmaster_01	Forward fall 45 °	p80	76.6	15.1	3.3	0.268
Centurion_Nexus_Heightmaster_02			76.7	15.3	3.7	0.272
Centurion_Nexus_Heightmaster_03			77.3	15.2	3.8	0.271
Mean			76.9	15.2	3.6	0.270
Standard Dev			0.3	0.1	0.2	0.002
Coefficient of Variance (%)			0	1	7	1
Kask_Superplasma_PL_01		Roof Shingle	70.2	15.0	2.6	0.265
Kask_Superplasma_PL_02			70.7	15.3	2.6	0.271
Kask_Superplasma_PL_03			73.8	15.3	2.9	0.272
Mean			71.6	15.2	2.7	0.269
Standard Dev			2.0	0.2	0.2	0.003
Coefficient of Variance (%)			3	1	8	1
Kask_Superplasma_PL_01	Forward fall	p80	137.2	5.6	2.4	0.101
Kask_Superplasma_PL_02			123.4	4.9	2.7	0.097
Kask_Superplasma_PL_03			134.9	6.4	2.9	0.112
Mean			131.8	5.6	2.7	0.103
Standard Dev			7.4	0.8	0.3	0.008
Coefficient of Variance (%)			6	14	10	8
Kask_Superplasma_PL_01		Roof Shingle	122.3	4.5	2.5	0.087
Kask_Superplasma_PL_02			122.7	4.2	2.1	0.078
Kask_Superplasma_PL_03			119.8	4.7	2.5	0.087
Mean			121.6	4.5	2.4	0.084
Standard Dev			1.6	0.3	0.2	0.005
Coefficient of Variance (%)			1	6	9	6
Kask_Superplasma_PL_01	Forward fall rim	p80	156.6	7.8	2.9	0.140
Kask_Superplasma_PL_02			142.9	7.4	2.5	0.134
Kask_Superplasma_PL_03			141.0	6.8	2.7	0.125
Mean			146.8	7.4	2.7	0.133
Standard Dev			8.5	0.5	0.2	0.008
Coefficient of Variance (%)			6	7	6	6
Kask_Superplasma_PL_01		Roof Shingle	174.4	8.5	3.0	0.152
Kask_Superplasma_PL_02			153.0	8.6	2.8	0.154
Kask_Superplasma_PL_03			150.3	8.1	2.7	0.148
Mean			159.2	8.4	2.8	0.151
Standard Dev			13.2	0.2	0.2	0.003
Coefficient of Variance (%)			8	3	7	2
Kask_Superplasma_PL_01	Rear fall	p80	138.6	2.9	2.0	0.062
Kask_Superplasma_PL_02			147.8	2.9	1.9	0.051
Kask_Superplasma_PL_03			144.9	2.9	1.9	0.061
Mean			143.8	2.9	2.0	0.058
Standard Dev			4.7	0.0	0.1	0.006
Coefficient of Variance (%)			3	1	3	10
Kask_Superplasma_PL_01		Roof Shingle	143.2	3.1	2.7	0.058
Kask_Superplasma_PL_02			143.5	3.3	2.5	0.059
Kask_Superplasma_PL_03			143.2	3.5	2.5	0.065
Mean			143.3	3.3	2.6	0.060
Standard Dev			0.2	0.2	0.1	0.004
Coefficient of Variance (%)			0	6	4	6
Kask_Superplasma_PL_01	Trip	p80	88.5	5.2	1.7	0.106
Kask_Superplasma_PL_02			85.0	4.5	1.3	0.106
Kask_Superplasma_PL_03			105.1	5.8	2.0	0.115
Mean			92.8	5.2	1.7	0.109
Standard Dev			10.7	0.7	0.4	0.005
Coefficient of Variance (%)			12	13	22	4
Kask_Superplasma_PL_01		Roof Shingle	80.0	4.4	1.7	0.104
Kask_Superplasma_PL_02			88.2	3.7	1.7	0.090
Kask_Superplasma_PL_03			74.3	5.9	1.9	0.112
Mean			80.8	4.7	1.8	0.102
Standard Dev			7.0	1.1	0.1	0.011
Coefficient of Variance (%)			9	23	5	11
MSA_V-Gard_01	Forward fall 45 °	p80	61.4	10.9	1.5	0.194
MSA_V-Gard_02			57.7	8.8	2.0	0.160
MSA_V-Gard_03			34.4	18.2	3.0	0.323
Mean			51.2	12.7	2.2	0.226
Standard Dev			14.6	4.9	0.8	0.086
Coefficient of Variance (%)			29	39	37	38
MSA_V-Gard_01		Roof Shingle	46.4	19.4	2.8	0.345
MSA_V-Gard_02			51.5	17.3	3.0	0.306
MSA_V-Gard_03			52.9	13.8	3.3	0.249
Mean			50.3	16.8	3.1	0.300
Standard Dev			3.4	2.8	0.3	0.049
Coefficient of Variance (%)			7	17	8	16
MSA_V-Gard_01	Forward fall	p80	113.1	15.1	2.9	0.268
MSA_V-Gard_02			113.1	15.6	2.3	0.279
MSA_V-Gard_03			107.0	15.7	3.5	0.278
Mean			111.1	15.5	2.9	0.275
Standard Dev			3.5	0.3	0.6	0.006
Coefficient of Variance (%)			3	2	21	2
MSA_V-Gard_01		Roof Shingle	108.7	13.8	3.0	0.245
MSA_V-Gard_02			166.4	15.0	2.9	0.264
MSA_V-Gard_03			110.2	16.6	3.4	0.328
Mean			128.4	15.1	3.1	0.279
Standard Dev			32.9	1.4	0.3	0.043
Coefficient of Variance (%)			26	9	9	16
MSA_V-Gard_01	Forward fall rim	p80	544.0	9.2	3.8	0.167
MSA_V-Gard_02			Not done due to high PLA in test 1			
MSA_V-Gard_03			Not done due to high PLA in test 1			
Mean			544.0	9.2	3.8	0.167
Standard Dev			0.0	0.0	0.0	0.000
Coefficient of Variance (%)			0	0	0	0
MSA_V-Gard_01	Rear fall	p80	484.9	3.3	1.5	0.062
MSA_V-Gard_02			113.5	6.8	3.7	0.133
MSA_V-Gard_03			130.5	5.5	3.5	0.134
Mean			243.0	2.9	5.2	0.110
Standard Dev			209.7	1.2	1.8	0.042
Coefficient of Variance (%)			86	42	34	38
MSA_V-Gard_01		Roof Shingle	115.4	7.0	4.0	0.126
MSA_V-Gard_02			277.8	6.7	3.2	0.125
MSA_V-Gard_03			115.2	5.6	2.1	0.112
Mean			169.4	6.4	3.1	0.121
Standard Dev			93.8	1.0	0.7	0.008
Coefficient of Variance (%)			55	30	12	7
MSA_V-Gard_01	Trip	p80	71.8	16.6	2.6	0.299
MSA_V-Gard_02			70.6	16.4	2.9	0.293
MSA_V-Gard_03			64.7	12.8	2.5	0.230
Mean			69.0	15.3	2.7	0.274
Standard Dev			3.8	2.1	0.2	0.038
Coefficient of Variance (%)			5	14	7	14
MSA_V-Gard_01		Roof Shingle	63.6	18.4	2.3	0.328
MSA_V-Gard_02			57.5	17.7	2.4	0.318
MSA_V-Gard_03			61.4	25.7	3.7	0.456
Mean			60.8	20.6	2.8	0.367
Standard Dev			3.1	4.4	0.8	0.077
Coefficient of Variance (%)			5	21	28	21
Centurion_Concept_Peak_Vented_01	Forward fall 45 °	p80	52.1	12.2	1.3	0.215
Centurion_Concept_Peak_Vented_02			54.3	12.0	1.3	0.212
Centurion_Concept_Peak_Vented_03			58.3	11.2	1.7	0.199
Mean			54.9	11.8	1.4	0.209
Standard Dev			3.1	0.5	0.3	0.009
Coefficient of Variance (%)			6	5	18	4
Centurion_Concept_Peak_Vented_01		Roof Shingle	57.2	11.2	1.4	0.199
Centurion_Concept_Peak_Vented_02			52.2	11.9	1.1	0.211
Centurion_Concept_Peak_Vented_03			55.5	11.7	1.5	0.208
Mean			55.0	11.6	1.3	0.206
Standard Dev			2.5	0.4	0.2	0.006
Coefficient of Variance (%)			5	3	14	3
Centurion_Concept_Peak_Vented_01	Forward fall	p80	106.2	5.4	1.4	0.108
Centurion_Concept_Peak_Vented_02			96.0	7.6	1.5	0.143
Centurion_Concept_Peak_Vented_03			97.8	9.2	1.4	0.184
Mean			100.0	7.4	1.4	0.145
Standard Dev			5.4	1.9	0.1	0.038
Coefficient of Variance (%)			5	26	4	26
Centurion_Concept_Peak_Vented_01		Roof Shingle	98.6	8.8	1.8	0.164
Centurion_Concept_Peak_Vented_02			95.0	9.2	1.4	0.204
Centurion_Concept_Peak_Vented_03			96.2	6.9	1.6	0.125
Mean			96.6	8.3	1.6	0.164
Standard Dev			1.8	1.2	0.2	0.040
Coefficient of Variance (%)			2	15	13	24
Centurion_Concept_Peak_Vented_01	Forward fall rim	p80	728.8	6.7	4.0	0.122
Centurion_Concept_Peak_Vented_02			Not done due to high PLA in test 1			
Centurion_Concept_Peak_Vented_03			Not done due to high PLA in test 1			
Mean			728.8	6.7	4.0	0.122
Standard Dev			0.0	0.0	0.0	0.000
Coefficient of Variance (%)			0	0	0	0
Centurion_Concept_Peak_Vented_01	Rear fall	p80	508.6	5.9	4.0	0.107
Centurion_Concept_Peak_Vented_02			281.9	3.1	2.9	0.063
Centurion_Concept_Peak_Vented_03			281.3	2.6	2.9	0.050
Mean			357.3	3.9	3.3	0.074
Standard Dev			131.1	1.8	0.6	0.030
Coefficient of Variance (%)			37	46	19	40
Centurion_Concept_Peak_Vented_01		Roof Shingle	392.0	4.1	4.2	0.075
Centurion_Concept_Peak_Vented_02			395.9	4.2	3.0	0.087
Centurion_Concept_Peak_Vented_03			429.0	4.4	4.0	0.085
Mean			405.6	4.2	3.7	0.082
Standard Dev			20.3	0.1	0.6	0.007
Coefficient of Variance (%)			5	3	17	8
Centurion_Concept_Peak_Vented_01	Trip	p80	77.5	8.3	1.1	0.168
Centurion_Concept_Peak_Vented_02			73.8	8.8	1.4	0.175
Centurion_Concept_Peak_Vented_03			70.5	9.6	1.8	0.177
Mean			73.9	8.9	1.4	0.173
Standard Dev			3.5	0.6	0.4	0.005
Coefficient of Variance (%)			5	7	26	3
Centurion_Concept_Peak_Vented_01		Roof Shingle	60.1	5.4	1.3	0.115
Centurion_Concept_Peak_Vented_02			68.3	10.5	2.1	0.191
Centurion_Concept_Peak_Vented_03			76.8	8.3	1.8	0.154
Mean			68.4	8.1	1.7	0.153
Standard Dev			8.4	2.6	0.4	0.038
Coefficient of Variance (%)			12	32	23	25
Uvex_Pheos_S-KR_01	Forward fall 45 °	p80	34.4	12.0	1.2	0.215
Uvex_Pheos_S-KR_02			35.0	12.2	1.2	0.218
Uvex_Pheos_S-KR_03			33.0	14.6	1.8	0.264
Mean			34.1	12.9	1.4	0.232
Standard Dev			1.0	1.4	0.3	0.027
Coefficient of Variance (%)			3	11	22	12
Uvex_Pheos_S-KR_01		Roof Shingle	37.8	14.4	1.3	0.257
Uvex_Pheos_S-KR_02			32.9	3.5	0.9	0.062
Uvex_Pheos_S-KR_03			33.1	3.8	1.3	0.075
Mean			34.6	7.2	1.2	0.131
Standard Dev			2.8	6.2	0.2	0.109
Coefficient of Variance (%)			8	86	20	83
Uvex_Pheos_S-KR_01	Forward fall	p80	154.3	7.0	2.2	0.134
Uvex_Pheos_S-KR_02			162.8	6.9	2.5	0.134
Uvex_Pheos_S-KR_03			165.4	6.8	2.4	0.127
Mean			160.8	6.9	2.4	0.132
Standard Dev			5.8	0.1	0.2	0.004
Coefficient of Variance (%)			4	1	6	3
Uvex_Pheos_S-KR_01		Roof Shingle	158.3	6.6	2.7	0.116
Uvex_Pheos_S-KR_02			146.4	8.4	3.1	0.155
Uvex_Pheos_S-KR_03			165.8	9.8	3.0	0.179
Mean			156.8	8.2	2.9	0.150
Standard Dev			9.8	1.6	0.2	0.032
Coefficient of Variance (%)			6	19	7	21
Uvex_Pheos_S-KR_01	Forward fall rim	p80	449.1	7.0	2.4	0.132
Uvex_Pheos_S-KR_02			Not done due to high PLA in test 1			
Uvex_Pheos_S-KR_03			Not done due to high PLA in test 1			
Mean			449.1	7.0	2.4	0.132
Standard Dev			0.0	0.0	0.0	0.000
Coefficient of Variance (%)			0	0	0	0
Uvex_Pheos_S-KR_01	Rear fall	p80	148.9	6.7	4.0	0.123
Uvex_Pheos_S-KR_02			139.4	7.4	4.1	0.130
Uvex_Pheos_S-KR_03			145.7	5.9	5.1	0.112
Mean			144.7	6.7	4.4	0.122
Standard Dev			4.9	0.7	0.6	0.009
Coefficient of Variance (%)			3	11	13	7
Uvex_Pheos_S-KR_01		Roof Shingle	142.7	7.5	4.0	0.134
Uvex_Pheos_S-KR_02			140.9	7.7	4.1	0.138
Uvex_Pheos_S-KR_03			156.6	8.3	4.1	0.148
Mean			146.7	7.9	4.1	0.140
Standard Dev			8.6	0.4	0.1	0.007
Coefficient of Variance (%)			6	5	2	5
Uvex_Pheos_S-KR_01	Trip	p80	76.4	8.3	1.4	0.169
Uvex_Pheos_S-KR_02			83.6	10.0	1.6	0.190
Uvex_Pheos_S-KR_03			79.8	8.7	1.8	0.156
Mean			80.0	9.0	1.6	0.172
Standard Dev			3.6	0.9	0.2	0.017
Coefficient of Variance (%)			4	10	14	10
Uvex_Pheos_S-KR_01		Roof Shingle	80.9	9.6	1.8	0.179
Uvex_Pheos_S-KR_02			93.2	9.6	1.5	0.171
Uvex_Pheos_S-KR_03			85.5	9.7	1.7	0.174
Mean			86.5	9.6	1.7	0.175
Standard Dev			6.2	0.1	0.1	0.004
Coefficient of Variance (%)			7	1	8	2
Uvex_Pheos_S-KR_MIPS_01	Forward fall 45 °	p80	41.5	12.3	0.9	0.216
Uvex_Pheos_S-KR_MIPS_02			38.2	11.8	1.3	0.209
Uvex_Pheos_S-KR_MIPS_03			38.2	10.8	0.6	0.194
Mean			39.3	11.7	0.9	0.206
Standard Dev			1.9	0.8	0.3	0.011
Coefficient of Variance (%)			5	7	38	5
Uvex_Pheos_S-KR_MIPS_01		Roof Shingle	39.7	10.1	0.7	0.178
Uvex_Pheos_S-KR_MIPS_02			40.3	10.0	0.6	0.178
Uvex_Pheos_S-KR_MIPS_03			37.6	11.1	1.1	0.196
Mean			39.2	10.4	0.8	0.184
Standard Dev			1.4	0.6	0.2	0.010
Coefficient of Variance (%)			4	6	31	6
Uvex_Pheos_S-KR_MIPS_01	Forward fall	p80	140.8	8.4	1.6	0.154
Uvex_Pheos_S-KR_MIPS_02			141.9	7.0	1.9	0.130
Uvex_Pheos_S-KR_MIPS_03			134.9	7.9	1.5	0.149
Mean			139.2	7.8	1.7	0.144
Standard Dev			3.8	0.7	0.2	0.013
Coefficient of Variance (%)			3	9	14	9
Uvex_Pheos_S-KR_MIPS_01		Roof Shingle	147.3	7.3	1.5	0.133
Uvex_Pheos_S-KR_MIPS_02			141.6	8.0	1.8	0.143
Uvex_Pheos_S-KR_MIPS_03			145.5	8.1	1.5	0.145
Mean			144.8	7.8	1.6	0.140
Standard Dev			2.9	0.4	0.1	0.007
Coefficient of Variance (%)			2	5	9	5
Uvex_Pheos_S-KR_MIPS_01	Forward fall rim	p80	495.0	7.6	2.2	0.143
Uvex_Pheos_S-KR_MIPS_02			Not done due to high PLA in test 1			
Uvex_Pheos_S-KR_MIPS_03			Not done due to high PLA in test 1			
Mean			495.0	7.6	2.2	0.143
Standard Dev			0.0	0.0	0.0	0.000
Coefficient of Variance (%)			0	0	0	0
Uvex_Pheos_S-KR_MIPS_01	Rear fall	p80	169.2	4.1	1.2	0.077
Uvex_Pheos_S-KR_MIPS_02			163.6	3.9	1.1	0.076
Uvex_Pheos_S-KR_MIPS_03			169.3	3.2	0.7	0.060
Mean			167.4	3.7	1.0	0.071
Standard Dev			3.3	0.4	0.2	0.009
Coefficient of Variance (%)			2	11	25	13
Uvex_Pheos_S-KR_MIPS_01		Roof Shingle	142.4	3.0	0.8	0.062
Uvex_Pheos_S-KR_MIPS_02			153.1	2.8	0.6	0.055
Uvex_Pheos_S-KR_MIPS_03			163.0	3.6	1.7	0.068
Mean			152.8	3.2	1.0	0.062
Standard Dev			10.3	0.4	0.6	0.007
Coefficient of Variance (%)			7	13	57	11
Uvex_Pheos_S-KR_MIPS_01	Trip	p80	76.0	8.1	1.2	0.162
Uvex_Pheos_S-KR_MIPS_02			73.2	8.6	1.2	0.162
Uvex_Pheos_S-KR_MIPS_03			76.0	8.6	1.5	0.152
Mean			75.1	8.4	1.3	0.159
Standard Dev			1.6	0.3	0.2	0.006
Coefficient of Variance (%)			2	4	17	3
Uvex_Pheos_S-KR_MIPS_01		Roof Shingle	63.4	7.5	1.5	0.149
Uvex_Pheos_S-KR_MIPS_02			69.1	9.6	1.5	0.172
Uvex_Pheos_S-KR_MIPS_03			73.2	9.3	1.5	0.169
Mean			68.6	8.8	1.5	0.163
Standard Dev			4.9	1.2	0.1	0.012
Coefficient of Variance (%)			7	13	3	8

These results are provided for both impact surfaces—P80 and roof shingle—and include the means and coefficients of variation for peak linear acceleration (PLA), peak rotational velocity (PRV), peak rotational acceleration (PRA), and the brain injury criterion (BrIC).
